# Supplementary material for: Projecting the Impact of Nutrition Policy to Improve Child Stunting: A Case Study in Guatemala Using the Lives Saved Tool
Source: Glob Health Sci Pract. 2021 Dec 31;9(4):752–64. doi: 10.9745/GHSP-D-20-00585 (PMC8691882; doi:10.9745/GHSP-D-20-00585)
Supplement: GHSP-D-20-00585-Supplement.pdf [file GHSP-D-20-00585-Supplement.pdf]

Supplement to: Tschida S, Cordon A, Asturias G, et al. Projecting the impact of nutrition policy to improve child stunting: a case study in Guatemala using the Lives Saved Tool. *Glob Health Sci Pract*. 2021;9(4). https://doi.org/10.9745/GHSP-D-20-00585

TABLE OF CONTENTS

INTERVENTIONS INCLUDED IN THE MODEL 3

MODEL INPUTS 4

Demographics 4
First year population single age 4
Total fertility rate 6
Age-specific fertility rate 7
Sex ratio at birth 8
Life expectancy 9
International migration 10

Health, Mortality and Economic Status 11
Baseline child health status 11
Meningococcal A vaccine recommended 11
Nutrition Deficiencies 11
Status at birth (percent of total births) 11
Incidence (Number of cases per child per year) 11
Nutrition status distributions 13
Stunting 13
Pathogens 14
Diarrhea – distribution among all cases 14
Diarrhea – distribution among severe cases 14
Diarrhea – distribution among fatal cases 14
Pneumonia – distribution among severe cases 14
Pneumonia – distribution among fatal cases 14
Meningitis – distribution among severe cases 15
Meningitis – distribution among fatal cases 15
Baseline child mortality 16
Mortality rate 16
Percent of child deaths by proximate cause 16
Household status 17
Poverty/food security 17

Intervention Coverages 18
Notes 18
Pregnancy 19
Nutritional 19
Breastfeeding 20
Breastfeeding Prevalence 20
Preventative 21
Food and supplementation 21
WASH (water, sanitation and hygiene) 21

Vaccines ... 22
Curative ... 23
Fertility Risks ... 24
Maternal age ... 24
Birth intervals ... 24

MODEL OUTPUTS ... **25**

Stunting Prevalence ... **25**

Stunting Cases Averted ... **26**

Stunting Cases Averted by Intervention ... **27**
Historical Trends ... 27
Great Crusade ... 28
Great Crusade (fertility) ... 29
Aspirational ... 30

INTERVENTIONS INCLUDED IN THE MODEL

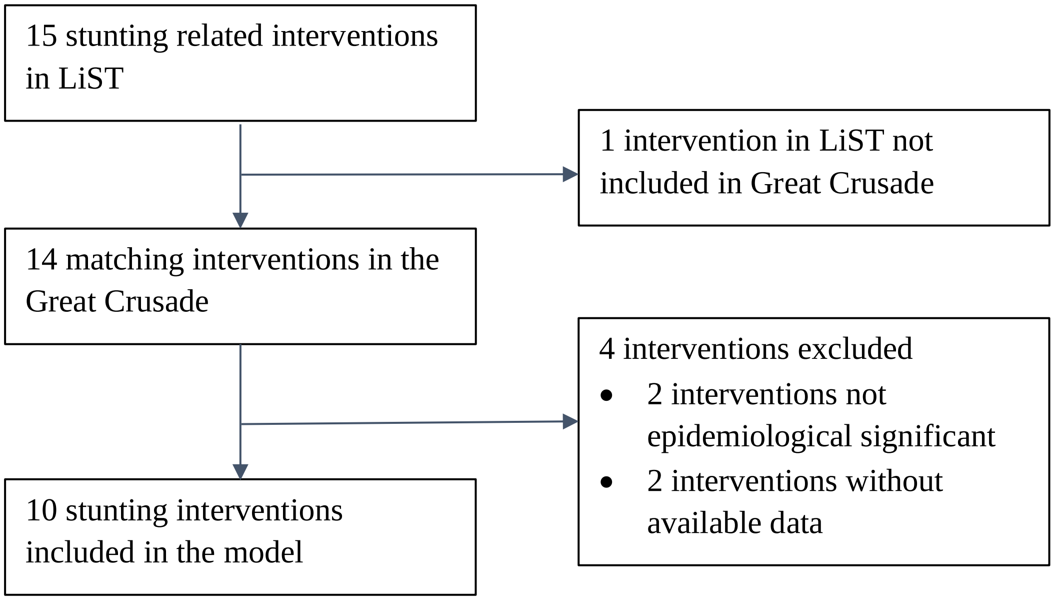

MODEL INPUTS

Demographics

First year population single age

Age	Male	Female		Age	Male	Female
**0**	193,140	184,360		**41**	87,311	97,985
**1**	193,084	184,827		**42**	83,725	95,009
**2**	193,486	185,541		**43**	80,193	92,056
**3**	192,847	184,749		**44**	76,729	89,056
**4**	191,977	183,962		**45**	73,357	85,974
**5**	191,156	183,246		**46**	70,112	82,802
**6**	190,401	182,608		**47**	66,987	79,328
**7**	188,802	181,171		**48**	63,960	75,996
**8**	186,540	179,120		**49**	61,013	72,636
**9**	185,852	178,580		**50**	58,159	69,338
**10**	183,284	176,249		**51**	55,397	66,175
**11**	182,564	175,701		**52**	52,767	63,202
**12**	181,812	175,138		**53**	50,260	60,413
**13**	181,017	174,554		**54**	47,895	57,801
**14**	180,174	173,973		**55**	45,699	55,357
**15**	179,270	173,387		**56**	43,737	53,440
**16**	178,264	172,757		**57**	41,949	51,298
**17**	177,084	172,004		**58**	40,261	49,220
**18**	182,554	177,758		**59**	38,640	47,185
**19**	185,290	180,995		**60**	37,047	45,184
**20**	183,174	179,629		**61**	35,475	43,235
**21**	175,274	172,717		**62**	33,937	41,329
**22**	170,249	168,605		**63**	32,441	39,478
**23**	165,229	164,481		**64**	30,997	37,685
**24**	162,470	162,513		**65**	29,603	35,944
**25**	159,398	160,214		**66**	28,280	34,255
**26**	155,974	157,453		**67**	27,022	32,624
**27**	152,121	154,213		**68**	25,821	31,032
**28**	147,939	150,618		**69**	24,654	29,469
**29**	143,401	146,664		**70**	23,426	27,881
**30**	138,517	142,395		**71**	22,346	26,413
**31**	133,356	137,920		**72**	21,259	24,963
**32**	128,035	133,330		**73**	20,142	23,507
**33**	122,667	128,704		**74**	18,985	22,028
**34**	117,383	124,132		**75**	17,793	20,531
**35**	112,281	119,709		**76**	16,569	19,046
**36**	107,450	115,479		**77**	15,346	17,601
**37**	102,928	111,485		**78**	14,133	16,212
**38**	98,734	107,772		**79**	12,943	14,884
**39**	94,718	104,258		**80+**	90,535	108,890

**40**	90,962	101,046		**Total**	8,297,763	8,560,479

Source: Estimaciones *y Proyecciones Nacionales de Población Metodología y Principales Resultados*. Guatemala City; 2019. (2019 Guatemalan Census)

Total fertility rate

	**2020**	**2021**	**2022**	**2023**	**2024**	**2025**	**2026**	**2027**	**2028**	**2029**	**2030**
**TFR**	2.6	2.5	2.4	2.4	2.3	2.3	2.2	2.2	2.2	2.1	2.1

Source: 2019 Guatemalan census

Age-specific fertility rate

Age	2020	2021	2022	2023	2024	2025	2026	2027	2028	2029	2030
**15-19**	12.17	12.12	12.08	12.02	11.90	11.90	11.83	11.76	11.68	11.60	11.51
**20-24**	24.27	24.24	24.21	24.18	24.10	24.10	24.06	24.01	23.96	23.90	23.85
**25-29**	23.55	23.58	23.61	23.64	23.73	23.73	23.77	23.82	23.87	23.93	23.99
**30-34**	19.38	19.42	19.46	19.52	19.64	19.64	19.71	19.78	19.87	19.96	20.05
**35-39**	13.08	13.09	13.10	13.12	13.16	13.16	13.18	13.21	13.23	13.26	13.28
**40-44**	6.10	6.09	6.08	6.07	6.05	6.05	6.03	6.02	6.00	5.99	5.97
**45-49**	1.47	1.46	1.45	1.44	1.42	1.42	1.41	1.40	1.38	1.37	1.36
**Total**	100.02	100	99.99	99.99	100	100	99.99	100	99.99	100.01	100.01

Source: Default data, World Population Prospects - Population Division - United Nations. https://population.un.org/wpp/

Sex ratio at birth

	**2020**	**2021**	**2022**	**2023**	**2024**	**2025**	**2026**	**2027**	**2028**	**2029**	**2030**
**Birth ratio**	103.7	103.7	103.7	103.7	103.7	103.7	103.7	103.7	103.7	103.7	103.7

Source: Calculated by authors using microdata from: International Ministerio de Salud Pública y Asistencia Social (MSPAS), Instituto Nacional de Estadística (INE), ICF International. *Encuesta Nacional de Salud Materno Infantil 2014-2015. Informe Final*.; 2017. (ENSMI)

Supplement to: Tschida S, Cordon A, Asturias G, et al. Projecting the impact of nutrition policy to improve child stunting: a case study in Guatemala using the Lives Saved Tool. *Glob Health Sci Pract*. 2021;9(4). https://doi.org/10.9745/GHSP-D-20-00585

Life expectancy

	2020	2021	2022	2023	2024	2025	2026	2027	2028	2029	2030
**Male**	69.9	70.1	70.3	70.5	70.7	70.8	71	71.2	71.4	71.6	71.8
**Female**	76.4	76.6	76.8	77	77.1	77.3	77.5	77.7	77.9	78	78.2

Source: 2019 Guatemalan census

International migration

	**2020**	**2021**	**2022**	**2023**	**2024**
**Male**	-21,098.20	-19,643.90	-18,346.30	-17,116.30	-16,030.90
**Female**	-17,876.60	-17,380.20	-16,827.10	-16,296.50	-15,711.40

	**2025**	**2026**	**2027**	**2028**	**2029**
**Male**	-15,153.70	-14,323.70	-13,608.60	-12,928.60	-12283.6
**Female**	-14,998.10	-14,317.80	-13,602.60	-12,922.40	-12277.2

	**2030**
**Male**	-11668.6
**Female**	-11662.1

Source 2019 Guatemalan census

Health, Mortality and Economic Status

Baseline child health status

Meningococcal A vaccine recommended

[ ] Yes [X] No

Nutrition Deficiencies

Input	Value	Source
**Percent vitamin A deficient**	0.0%	Instituto de Nutrición de Centro América y Panamá (INCAP). Sistema de Vigilancia Epidemiológica de Salud y Nutrición (SIVESNU). Guatemala: INCAP; 2016
**Percent zinc deficient**	13.3%	SIVESNU 2016

Status at birth (percent of total births)

Input	Value	Source
**Preterm: Small for gestational age (PEG)**	1.61%	Default data<br><br>Lee AC, Katz J, Blencowe H, et al. National and regional estimates of term and preterm babies born small for gestational age in 138 low-income and middle-income countries in 2010. Lancet Global Health 2013; 1(1): e26-36. http://www.ncbi.nlm.nih.gov/pubmed/25103583.
**Preterm: Appropriate for gestational age (AEG)**	6.10%	
**Term: Small for gestational age (PEG)**	11.96%	
**Term: Appropriate for gestational age (AEG)**	80.33%	
**Total**	100%	

Incidence (Number of cases per child per year)

	**< 1 month**	**1-5 months**	**6-11 months**	**12-23 months**	**24-59 months**	**Source**
**Incidence of diarrhea**	3.4	3.2	3.2	3.2	3.2	Default data<br><br>Fischer Walker CL, Rudan I, Liu L, et al. Global burden of childhood pneumonia and diarrhoea. The Lancet 2013; 381(9875): 1405-16. http://www.ncbi.nlm.nih.gov/pubmed/23582727.
**Incidence of severe diarrhea**	0.062	0.062	0.062	0.062	0.062	
**Incidence of severe pneumo-nia**	0.027	0.027	0.027	0.027	0.027	Default data<br><br>Rudan I, O'Brien KL, Nair H, et al. Epidemiology and etiology of childhood

	0.001	0.001	0.001	0.001	0.001	pneumonia in 2010: Estimates of incidence, severe morbidity, mortality, underlying risk factors and causative pathogens for 192 countries. Journal of Global Health 2013; 3(1). http://www.ncbi.nlm.nih.gov/pubmed/23826505. (Online supplementary material.)
**Incidence of meningit-is**	0.001	0.001	0.001	0.001	0.001	Default data<br><br>Calculated from the following sources:<br><br>Davis S, Feikin D, Johnson HL. The effect of Haemophilus influenzae type B and pneumococcal conjugate vaccines on childhood meningitis mortality: A systematic review. BMC Public Health 2013; 13(Suppl 3): S21. http://www.ncbi.nlm.nih.gov/pubmed/24564188.<br><br>See also the WHO online companion table referenced in the following articles: Watt JP, Wolfson LJ, O'Brien KL, et al. Burden of disease caused by Haemophilus influenzae type b in children younger than 5 years: Global estimates. Lancet 2009; 374(9693): 903-911. http://www.ncbi.nlm.nih.gov/pubmed/19748399.<br><br>O'Brien KL, Wolfson LJ, Watt JP, et al. Burden of disease caused by Streptococcus pneumoniae in children younger than 5 years: Global estimates. Lancet 2009; 374(9693): 893-902. http://www.ncbi.nlm.nih.gov/pubmed/19748398.

Nutrition status distributions

Stunting

	**Percent**	**Source**
**Stunting**	46.47%	Automatically calculated from stunting distribution.

**Stunting distribution**	**<1 month**	**1-5 months**	**6-11 months**	**12-23 months**	**24-59 months**	**Source**
**Greater than 1 SD less than the median norm**	36.03%	36.03%	31.34%	19.07%	19.84%	Calculated by authors using microdata from ENSMI 2014-2015.
**Between 1 and 2 SDs less than the median norm**	34.01%	34.01%	34.24%	30.16%	30.37%	
**Between 2 and 3 SDs less than the median norm**	22.49%	22.49%	23.94%	29.07%	31.94%	
**More than 3 standard deviations less than the median norm**	7.466%	7.466%	10.49%	21.7%	17.85%	
**Total**	100.00%	100.00%	100.01%	100.00%	100.00%	

Pathogens

Diarrhea – distribution among all cases

	1-5 months	6-11 months	12-23 months	24-59 months
**Rotavirus**	5.5%	5.5%	5.5%	5.5%
**Pathogen B**	0%	0%	0%	0%
**Pathogen C**	0%	0%	0%	0%
**All other pathogens**	94.5%	94.5%	94.5%	94.5%
**Total**	100%	100%%	100%	100%

Diarrhea – distribution among severe cases

	1-5 months	6-11 months	12-23 months	24-59 months
**Rotavirus**	0%	0%	0%	0%
**Pathogen B**	23.4%	23.4%	23.4%	23.4%
**Pathogen C**	0%	0%	0%	0%
**All other pathogens**	76.6%	76.6%	76.6%	76.6%
**Total**	100%	100%	100%	100%

Diarrhea – distribution among fatal cases

	1-5 months	6-11 months	12-23 months	24-59 months
**Rotavirus**	23.4%	23.4%	23.4%	23.4%
**Pathogen B**	0%	0%	0%	0%
**Pathogen C**	0%	0%	0%	0%
**All other pathogens**	76.6%	76.6%	76.6%	76.6%
**Total**	100%	100%	100%	100%

Source: Default data, Fischer Walker CL, Rudan I, Liu L, et al. Global burden of childhood pneumonia and diarrhoea. The Lancet 2013; 381(9875): 1405-16. http://www.ncbi.nlm.nih.gov/pubmed/23582727

Pneumonia – distribution among severe cases

	1-5 months	6-11 months	12-23 months	24-59 months
**H. influenzae type b**	4%	4%	4%	4%
**S. pneumoniae**	7%	7%	7%	7%
**Influenza virus**	10.7%	10.7%	10.7%	10.7%
**All other pathogens**	78.3%	78.3%	78.3%	78.3%
**Total**	100%	100%	100%	100%

Pneumonia – distribution among fatal cases

	1-5 months	6-11 months	12-23 months	24-59 months

**H. influenzae type b**	21.3%	21.3%	21.3%	21.3%
**S. pneumoniae**	32.8%	32.8%	32.8%	32.8%
**Influenza virus**	10.7%	10.7%	10.7%	10.7%
**All other pathogens**	35.2%	35.2%	35.2%	35.2%
**Total**	100%	100%	100%	100%

Source: Default data, Rudan I, O'Brien KL, Nair H, et al. Epidemiology and etiology of childhood pneumonia in 2010: Estimates of incidence, severe morbidity, mortality, underlying risk factors and causative pathogens for 192 countries. Journal of Global Health 2013; 3(1). http://www.ncbi.nlm.nih.gov/pubmed/23826505. Please refer to the online supplementary document

Meningitis – distribution among severe cases

	**1-5 months**	**6-11 months**	**12-23 months**	**24-59 months**
**H. influenzae type b**	46%	46%	46%	46%
**S. pneumoniae**	52%	52%	52%	52%
**N. meningitis type A**	2%	2%	2%	2%
**All other pathogens**	0%	0%	0%	0%
**Total**	100%	100%	100%	100%

Meningitis – distribution among fatal cases

	**1-5 months**	**6-11 months**	**12-23 months**	**24-59 months**
**H. influenzae type b**	46%	46%	46%	46%
**S. pneumoniae**	52%	52%	52%	52%
**N. meningitis type A**	2%	2%	2%	2%
**All other pathogens**	0%	0%	0%	0%
**Total**	100%	100%	100%	100%

Source: Default data, Davis S, Feikin D, Johnson HL. The effect of Haemophilus influenzae type B and pneumococcal conjugate vaccines on childhood meningitis mortality: A systematic review. BMC Public Health 2013; 13(Suppl 3): S21. http://www.ncbi.nlm.nih.gov/pmc/articles/PMC3847464/

Supplement to: Tschida S, Cordon A, Asturias G, et al. Projecting the impact of nutrition policy to improve child stunting: a case study in Guatemala using the Lives Saved Tool. *Glob Health Sci Pract*. 2021;9(4). https://doi.org/10.9745/GHSP-D-20-00585

Baseline child mortality

Mortality rate

	**Mortality rate**
**Neonatal**	11.58
**Infant**	20.56
**Child**	24.19

Source: Calculated by authors using historical data from: UN Inter-agency Group for Child Mortality Estimation. CME info - Child Mortality Estimates. http://childmortality.org.

Percent of child deaths by proximate cause

**Neonatal**	**Percent**	**Post neonatal**	**Percent**
**Diarrhea**	0.4%	**Diarrhea**	13.1%
**Sepsis**	16.8%	**Pneumonia**	23.5%
**Pneumonia**	5.9%	**Meningitis**	1.9%
**Asphyxia**	25.0%	**Measles**	0.0%
**Prematurity**	27.2%	**Malaria**	0.0%
**Tetanus**	0.0%	**Pertussis**	1.0%
**Congenital anomalies**	16.5%	**AIDS**	1.2%
**Other**	8.3%	**Injury**	16.7%
**Total**	100.0%	**Other**	42.6%
		**Total**	100.0%

Source: Default data, WHO estimates for years 2000-2017.
https://www.who.int/healthinfo/global_burden_disease/estimates/en/index2.html

Liu L, Oza S, Hogan D, et al. Global, regional, and national causes of under-5 mortality in 2000-15: an updated systematic analysis with implications for the Sustainable Development Goals. Lancet 2016.
https://www.ncbi.nlm.nih.gov/pubmed/27839855

Household status

Poverty/food security

	**2020**	**2021**	**2022**	**2023**	**2024**	**2025**	**2026**	**2027**	**2028**	**2029**	**2030**
**Percentage of the population that is food insecure**	22.6%	22.6%	22.6%	22.6%	22.6%	22.6%	22.6%	22.6%	22.6%	22.6%	22.6%

Source: SIVESNU 2015

Intervention Coverages

Notes

- Abbreviations
    - HT = Historical trends
    - GC = Great Crusade
- Coverage levels in the Great Crusade model remain static after 2024.
- The Great Crusade fertility and aspirational model are not included in the tables for clarity.
    - The Great Crusade fertility model has the same coverages as the Great Crusade model.
    - Coverage levels in the Aspirational model reach 90.0% in 2024 and remain static till 2030.
- Coverages are linearly interpolated.

Pregnancy

Nutritional

Input	Baseline (2020)	HT (2024)	HT (2030)	GC (2024)	Source
**Calcium supplementation in pregnancy**	No data				
**Multiple micronutrient supplementation in pregnancy**	82.3%	82.3%	82.3%	90.0%	SIVESNU 2018

Breastfeeding

Breastfeeding Prevalence

Input	Baseline (2020)	HT (2024)	HT (2030)	GC (2024)	Source
**Prevalence of early initiation of breastfeeding**	66.2%	68.6%	72.0%	71.2%	Projected value calculated by authors using historical ENSMI data.

	<1 month (2020)	HT (2024)	HT (2030)	GC (2024)
**Exclusive**	63.7%	65.1%	68.4%	67.7%
**Predominant**	13.5%	12.9%	11.5%	12.3%
**Partial**	19.1%	18.5%	17.1%	17.4%
**None**	3.7%	3.5%	3.0%	2.6%
**Total**	100.0%	100.0%	100.0%	100.0%

	1-5 months (2020)	HT (2024)	HT (2030)	GC 2024)
**Exclusive**	52%	53.9%	56.7%	56.0%
**Predominant**	16.9%	14.7%	14.7%	15.0%
**Partial**	25.8%	23.5%	23.5%	23.9%
**None**	5.3%	5.1%	5.1%	5.1%
**Total**	100.0%	100.0%	100.0%	100.0%

	6-11 months (2020)	HT (2024)	HT (2030)	GC (2024)
**Any type**	90.0%	90.0%	90.0%	90.0%
**None**	10.0%	10.0%	10.0%	10.0%
**Total**	100.0%	100.0%	100.0%	100.0%

	12-23 months (2020)	HT (2024)	HT (2030)	GC (2024)
**Any type**	71.3%	71.6%	72.1%	71.6%
**None**	28.7%	28.4%	27.9%	28.4%
**Total**	100.0%	100.0%	100.0%	100.0%

Source: Projected value calculated by authors using historical ENSMI data.

Preventative

Food and supplementation

Input	Baseline (2020)	HT (2024)	HT (2030)	GC (2024)	Source
**Comp. feeding (only education)**	62.6%	62.6%	62.6%	72.6%	ENSMI 2014-2015
**Comp. feeding (feeding & education)**	62.6%	62.6%	62.%	72.6%	ENSMI 2014-2015
**Vitamin A supplementation for children**	26.0%	26.0%	26.0%	51.0%	Vitamin A Deficiency in Children - UNICEF Data.https://data.unicef.org/topic/nutrition/vitamin-a-deficiency/. Published 2019
**Zinc supplementation for children**	86.1%	86.1%	86.1%	90.0%	SIVESNU 2018

WASH (water, sanitation and hygiene)

Input	Baseline (2020)	HT (2024)	HT (2030)	GC (2024)	Source
**Basic sanitation**	65.4%	65.7%	66.2%	90.0%	Projected value calculated by authors using historical data from: WHO/UNICEF Joint Monitoring Program (JMP) for Water Supply and Sanitation (https://washdata.org/). Data are available for all countries from 1996 to 2015.
**Point-of-use water filter or piped in water**	89.2%	91.4%	93.9%	91.4%	
**Handwashing with soap**	76.9%	77.1%	77.4%	90.0%	

Vaccines

Input	Baseline (2020)	HT (2024)	HT (2030)	GC (2024)	Source
**Rotavirus (2 doses)**	88.4%	90.0%	90.0%	90.0%	Projected value calculated by authors using historical data from ENSMI.

Curative

**Input**	**Baseline (2020)**	**Source**
**Kangaroo mother care (KMC)**	No data	

Fertility Risks

Maternal age

Less than 18 years old	2020*	2030*	GC (fertility) 2030
First birth	8.0%	7.8%	0.0%
Second and third birth	0.9%	0.6%	0.0%
Greater than third birth	0.0%	0.0%	0.0%
**18 to 34 years old**			
First birth	26.5%	32.1%	39.9%
Second and third birth	39.5%	39.2%	39.8%
Greater than third birth	14.5%	9.1%	9.1%
**35 to 49 years old**			
First birth	0.5%	0.9%	0.9%
Second and third birth	3.3%	5.9%	5.9%
Greater than third birth	6.8%	4.4%	4.4%
**Total**	100%	100%	100.0%

Source: Calculated by authors using microdata from ENSMI 2014-2015.

Birth intervals

Birth intervals	2020*	2030*	GC (fertility) 2030
First birth	35.4%	43.3%	43.3%
Less than 18 months	3.0%	2.0%	0.0%
18-23 months	6.6%	4.3%	0.0%
24 months or more	55.0%	50.4%	56.7%
Total	100.0%	100%	100%

Source: Calculated by authors using microdata from ENSMI 2014-2015.

*Note: The historical trends, Great Crusade, and aspirational models have the same fertility risk.

MODEL OUTPUTS

Stunting Prevalence

**Stunting Prevalence Median % (95% CI)**	**2020**	**2021**	**2022**	**2023**	**2024**	**2025**	**2026**	**2027**	**2028**	**2029**	**2030**
**Historical Trends**	46.48 (46.48 - 46.48)	46.51 (46.49 - 46.52)	46.51 (46.46 - 46.55)	46.38 (46.3 - 46.43)	46.39 (46.26 - 46.47)	46.25 (46.08 - 46.36)	46.29 (46.08 - 46.43)	46.19 (45.94 - 46.36)	46.11 (45.81 - 46.3)	46.18 (45.84 - 46.4)	46.09 (45.71 - 46.33)
**Great Crusade**	46.48 (46.48 - 46.48)	46.37 (46.3 - 46.42)	46.16 (45.99 - 46.27)	45.75 (45.46 - 45.93)	45.41 (44.98 - 45.68)	45.03 (44.5 - 45.37)	44.91 (44.3 - 45.3)	44.72 (44.06 - 45.15)	44.62 (43.93 - 45.07)	44.71 (43.99 - 45.17)	44.63 (43.89 - 45.11)
**Great Crusade (fertility)**	46.48 (46.48 - 46.48)	46.37 (46.29 - 46.42)	46.16 (45.96 - 46.28)	45.74 (45.4 - 45.95)	45.39 (44.88 - 45.71)	45.01 (44.37 - 45.41)	44.87 (44.13 - 45.34)	44.68 (43.86 - 45.2)	44.58 (43.69 - 45.13)	44.66 (43.71 - 45.24)	44.57 (43.57 - 45.18)
**Aspirational**	46.48 (46.48 - 46.48)	46.25 (46.12 - 46.33)	45.81 (45.49 - 46.02)	45.06 (44.51 - 45.44)	44.28 (43.45 - 44.85)	43.56 (42.52 - 44.28)	43.19 (42.01 - 44.02)	42.88 (41.61 - 43.78)	42.75 (41.44 - 43.68)	42.83 (41.5 - 43.77)	42.76 (41.41 - 43.71)

Stunting Cases Averted

Stunting Cases Averted	2020	2021	2022	2023	2024	2025	2026	2027	2028	2029	2030
**Historical Trends**	0 (0 - 0)	328 (126 - 680)	757 (228 - 1,590)	1,381 (457 - 2,803)	1,997 (646 - 4,196)	2,640 (766 - 5,659)	3,307 (924 - 7,087)	3,912 (1,038 - 8,418)	4,479 (1,148 - 9,662)	5,010 (1,272 - 10,836)	5,496 (1,362 - 11,934)
**Great Crusade**	0 (0 - 0)	2,938 (2,042 - 4,290)	7,254 (5,128 - 10,384)	12,878 (9,246 - 18,153)	19,684 (14,292 - 27,303)	24,308 (17,683 - 33,587)	27,584 (20,082 - 38,095)	29,551 (21,413 - 40,947)	30,178 (2,1691 - 42,078)	30,345 (21,663 - 42,546)	30,313 (21,466 - 42,772)
**Great Crusade (fertility)**	0 (0 - 0)	2,973 (2,000 - 4,462)	7,324 (5,022 - 10,767)	13,027 (9,041 - 18,820)	19,884 (13,987 - 28,356)	24,637 (17,254 - 35,116)	28,038 (19,538 - 40,140)	30,098 (20,724 - 43,549)	30,853 (20,838 - 45,302)	31,120 (20,648 - 46,349)	31,213 (20,277 - 47,222)
**Aspirational**	0 (0 - 0)	5,254 (3,572 - 7,631)	13,760 (9,598 - 19,489)	25,324 (17,975 - 35,240)	39,632 (28,567 - 54,259)	49,992 (36,174 - 68,041)	57,128 (41,473 - 77,443)	61,107 (44,284 - 82,853)	62,067 (44,754 - 84,412)	62,069 (44,665 - 84,557)	61,625 (44,172 - 84,193)

Stunting Cases Averted by Intervention

Historical Trends

Stunting Cases Averted	2020	2021	2022	2023	2024	2025	2026	2027	2028	2029	2030
**Birth intervals**	0 (0 - 0)	0 (0 - 62)	9 (0 - 161)	17 (0 - 281)	30 (0 - 466)	44 (0 - 761)	54 (0 - 939)	66 (0 - 1,140)	80 (0 - 1,261)	91 (0 - 1,432)	101 (0 - 1,609)
**Breastfeeding**	0 (0 - 0)	16 (3 - 52)	42 (2 - 128)	95 (8 - 254)	144 (10 - 386)	217 (14 - 563)	266 (16 - 696)	332 (19 - 867)	407 (26 - 1,059)	440 (24 - 1,154)	499 (27 - 1,304)
**Filtered water/piped in water**	0 (0 - 0)	312 (155 - 563)	677 (275 - 1,240)	1,218 (500 - 2,167)	1,728 (703 - 3,157)	2,253 (858 - 4,091)	2,812 (1,024 - 5,112)	3,310 (1,164 - 6,012)	3,761 (1,284 - 6,886)	4,200 (1,423 - 7,697)	4,594 (1,547 - 8,422)
**Basic sanitation**	0 (0 - 0)	0 (0 - 0)	15 (5 - 28)	35 (13 - 65)	58 (21 - 109)	86 (29 - 160)	113 (36 - 210)	141 (44 - 262)	144 (44 - 269)	170 (51 - 319)	194 (58 - 365)
**Hand washing with soap**	0 (0 - 0)	0 (0 - 0)	14 (5 - 29)	16 (6 - 33)	36 (13 - 74)	40 (14 - 82)	63 (21 - 128)	65 (21 - 134)	89 (27 - 183)	113 (34 - 232)	113 (34 - 232)
**Rotavirus vaccine**	0 (0 - 0)	3 (2 - 3)	3 (2 - 3)	3 (2 - 3)	2 (2 - 3)	2 (2 - 3)	2 (1 - 3)	2 (1 - 3)	2 (1 - 3)	2 (1 - 3)	2 (1 - 3)

Great Crusade

Stunting Cases Averted	2020	2021	2022	2023	2024	2025	2026	2027	2028	2029	2030
**Micronutrient supplementation (iron and multiple micronutrients)**	0 (0 - 0)	17 (8 - 30)	48 (26 - 83)	89 (49 - 152)	138 (78 - 232)	178 (102 - 293)	194 (111 - 319)	203 (116 - 336)	204 (116 - 340)	200 (113 - 333)	197 (109 - 329)
**Birth intervals**	0 (0 - 0)	6 (0 - 69)	14 (0 - 176)	23 (0 - 306)	39 (0 - 496)	63 (0 - 901)	80 (0 - 1,143)	96 (0 - 1,375)	113 (0 - 1,471)	124 (0 - 1,624)	134 (0 - 1,786)
**Breastfeeding**	0 (0 - 0)	45 (4 - 104)	121 (7 - 279)	223 (14 - 505)	363 (36 - 789)	467 (45 - 997)	512 (51 - 1,089)	538 (54 - 1,144)	540 (54 - 1,155)	530 (53 - 1,132)	520 (51 - 1,114)
**Complementary feeding**	0 (0 - 0)	758 (737 - 766)	2,199 (2,147 - 2,222)	4,340 (4,241 - 4,389)	7,172 (6,997 - 7,266)	9,214 (9,011 - 9,313)	10,640 (10,380 - 10,757)	11,389 (11,061 - 11,527)	11,532 (11,113 - 11,698)	11,455 (10,981 - 11,625)	11,265 (10,745 - 11,441)
**Zinc supplementation**	0 (0 - 0)	438 (411 - 478)	924 (865 - 1,004)	1,482 (1,376 - 1,602)	2,105 (1,948 - 2,278)	2,257 (2,084 - 2,451)	2,423 (2,228 - 2,632)	2,509 (2,298 - 2,728)	2,503 (2,277 - 2,725)	2,488 (2,253 - 2,714)	2,454 (2,213 - 2,677)
**Filtered water/piped in water**	0 (0 - 0)	350 (201 - 567)	764 (445 - 1,230)	1,334 (781 - 2,132)	1,905 (1,121 - 3,028)	2,668 (1,569 - 4,204)	3,452 (2,030 - 5,436)	4,069 (2,385 - 6,405)	4,536 (2,644 - 7,166)	4,938 (2,866 - 7,799)	5,308 (3,064 - 8,387)
**Basic sanitation**	0 (0 - 0)	885 (449 - 1,467)	2,116 (1,093 - 3,476)	3,590 (1,870 - 5,861)	5,291 (2,773 - 8,598)	6,281 (3,300 - 10,102)	6,822 (3,584 - 10,960)	7,124 (3,730 - 11,438)	7,128 (3,712 - 11,485)	7,036 (3,648 - 11,340)	6,920 (3,567 - 11,156)
**Hand washing with soap**	0 (0 - 0)	437 (229 - 805)	1,044 (551 - 1,898)	1,772 (937 - 3,193)	2,611 (1,385 - 4,676)	3,097 (1,639 - 5,484)	3,364 (1,778 - 5,952)	3,513 (1,850 - 6,212)	3,515 (1,840 - 6,237)	3,470 (1,809 - 6,159)	3,412 (1,769 - 6,059)
**Rotavirus vaccine**	0 (0 - 0)	3 (2 - 3)	3 (2 - 3)	2 (2 - 3)	2 (2 - 3)	2 (2 - 3)	2 (2 - 3)	2 (2 - 3)	2 (2 - 3)	2 (2 - 2)	2 (2 - 2)

Great Crusade (fertility)

Stunting Cases Averted	2020	2021	2022	2023	2024	2025	2026	2027	2028	2029	2030
**Micronutrient supplementation (iron and multiple micronutrients)**	0 (0 - 0)	9 (3 - 15)	25 (11 - 42)	46 (22 - 76)	71 (36 - 116)	91 (47 - 145)	99 (51 - 157)	104 (53 - 165)	105 (52 - 166)	103 (51 - 163)	101 (49 - 161)
**Maternal age and birth order**	0 (0 - 0)	14 (8 - 15)	41 (28 - 42)	75 (56 - 76)	118 (92 - 119)	183 (150 - 186)	247 (204 - 254)	300 (244 - 309)	344 (273 - 356)	375 (296 - 388)	409 (319 - 425)
**Birth intervals**	0 (0 - 0)	17 (0 - 235)	48 (0 - 650)	88 (0 - 1191)	138 (0 - 1835)	224 (0 - 2906)	298 (0 - 3821)	369 (0 - 4722)	431 (0 - 5512)	482 (0 - 6173)	536 (0 - 6867)
**Breastfeeding**	0 (0 - 0)	46 (4 - 105)	123 (7 - 277)	226 (14 - 500)	366 (34 - 778)	470 (43 - 974)	516 (48 - 1,060)	542 (50 - 1,110)	546 (49 - 1,118)	536 (48 - 1,099)	527 (47 - 1,084)
**Complementary feeding**	0 (0 - 0)	761 (725 - 767)	2,196 (2,096 - 2,220)	4,337 (4,134 - 4,380)	7,169 (6,820 - 7,251)	9,170 (8,739 - 9,291)	10,571 (10,013 - 10,719)	11,302 (10,620 - 11,477)	11,441 (10,609 - 11,615)	11,377 (10,411 - 11,558)	11,211 (10,108 - 11,379)
**Zinc supplementation**	0 (0 - 0)	438 (411 - 478)	924 (863 - 1,001)	1,482 (1,364 - 1,598)	2,107 (1,919 - 2,281)	2,260 (2,042 - 2,453)	2,425 (2,176 - 2,627)	2,514 (2,237 - 2,716)	2,510 (2,208 - 2,712)	2,498 (2,172 - 2,699)	2,466 (2,121 - 2,666)
**Filtered water/piped in water**	0 (0 - 0)	352 (195 - 568)	767 (431 - 1,221)	1,340 (754 - 2,107)	1,911 (1,083 - 2,985)	2,678 (1,510 - 4,120)	3,467 (1,946 - 5,305)	4,084 (2,268 - 6,235)	4,558 (2,490 - 6,961)	4,965 (2,685 - 7,588)	5,345 (2,853 - 8,178)
**Basic sanitation**	0 (0 - 0)	891 (433 - 1,470)	2,124 (1,055 - 3,450)	3,606 (1,801 - 5,790)	5,309 (2,674 - 8,470)	6,301 (3,170 - 9,901)	6,846 (3,429 - 10,708)	7,144 (3,541 - 11,151)	7,157 (3,488 - 11,174)	7,071 (3,411 - 11,050)	6,964 (3,315 - 10,894)
**Hand washing with soap**	0 (0 - 0)	440 (221 - 806)	1,049 (533 - 1,884)	1,780 (905 - 3,155)	2,619 (1,338 - 4,608)	3,109 (1,577 - 5,373)	3,378 (1,703 - 5,808)	3,525 (1,758 - 6,046)	3,531 (1,732 - 6,058)	3,489 (1,694 - 5,991)	3,436 (1,647 - 5,906)
**Rotavirus vaccine**	0 (0 - 0)	3 (2 - 3)	3 (2 - 3)	2 (2 - 3)	2 (2 - 3)	2 (2 - 3)	2 (2 - 3)	2 (2 - 3)	2 (2 - 2)	2 (2 - 2)	2 (2 - 2)

Aspirational

Stunting Cases Averted	2020	2021	2022	2023	2024	2025	2026	2027	2028	2029	2030
**Micronutrient supplementation (iron and multiple micronutrients)**	0 (0 - 0)	11 (0 - 20)	30 (12 - 53)	55 (24 - 96)	83 (39 - 145)	106 (53 - 183)	115 (58 - 198)	121 (61 - 207)	121 (61 - 209)	119 (60 - 205)	117 (58 - 202)
**Birth intervals**	0 (0 - 0)	7 (0 - 90)	17 (0 - 222)	28 (0 - 381)	46 (0 - 608)	74 (0 - 1,096)	94 (0 - 1,384)	112 (0 - 1,660)	132 (0 - 1,771)	145 (0 - 1,953)	158 (0 - 2,148)
**Breastfeeding**	0 (0 - 0)	638 (121 - 1,416)	1,650 (281 - 3,602)	3,008 (526 - 6,425)	4,642 (853 - 9,695)	5,820 (1,008 - 12,077)	6,550 (1,193 - 13,464)	6,936 (1,281 - 14,201)	6,979 (1,291 - 14,305)	6,887 (1,287 - 14,074)	6,766 (1,263 - 13,813)
**Complementary feeding**	0 (0 - 0)	2,337 (2,151 - 2,479)	6,739 (6,247 - 7,074)	13,239 (12,278 - 13,912)	21,819 (20,181 - 22,987)	28,230 (26,136 - 29,564)	32,666 (30,148 - 34,167)	34,976 (32,187 - 36,520)	35,358 (32,418 - 37,022)	35,154 (32,187 - 36,812)	34,611 (31,645 - 36,224)
**Zinc supplementation**	0 (0 - 0)	451 (418 - 499)	989 (905 - 1,099)	1,612 (1,444 - 1,809)	2,304 (2,044 - 2,601)	2,492 (2,191 - 2,826)	2,682 (2,346 - 3,044)	2,782 (2,426 - 3,158)	2,773 (2,410 - 3,150)	2,760 (2,395 - 3,135)	2,725 (2,363 - 3,094)
**Filtered water/piped in water**	0 (0 - 0)	378 (202 - 626)	841 (458 - 1,382)	1,470 (808 - 2,410)	2,081 (1,158 - 3,388)	2,928 (1,639 - 4,737)	3,789 (2,127 - 6,121)	4,463 (2,502 - 7,199)	4,972 (2,776 - 8,031)	5,415 (3,022 - 8,731)	5,829 (3,242 - 9,386)
**Basic sanitation**	0 (0 - 0)	951 (447 - 1,605)	2,329 (1,122 - 3,906)	3,949 (1,926 - 6,605)	5,782 (2,861 - 9,607)	6,895 (3,445 - 11,382)	7,488 (3,754 - 12,351)	7,813 (3,912 - 12,873)	7,814 (3,897 - 12,889)	7,717 (3,845 - 12,711)	7,600 (3,774 - 12,501)
**Hand washing with soap**	0 (0 - 0)	477 (231 - 893)	1,158 (571 - 2,147)	1,949 (967 - 3,598)	2,852 (1,430 - 5,225)	3,399 (1,711 - 6,174)	3,691 (1,862 - 6,696)	3,852 (1,940 - 6,977)	3,852 (1,932 - 6,984)	3,805 (1,906 - 6,888)	3,747 (1,872 - 6,775)
**Rotavirus vaccine**	0 (0 - 0)	3 (2 - 3)	3 (2 - 3)	3 (2 - 3)	3 (2 - 3)	3 (2 - 3)	3 (2 - 3)	3 (2 - 3)	2 (2 - 3)	2 (2 - 3)	2 (2 - 3)
